# Estimation of the poliovirus type 2 immunity gap in South Africa

**DOI:** 10.1016/j.vaccine.2024.06.029

**Published:** 2024-10-03

**Authors:** Lauren Brown, Jeremy Bingham, Juliet Pulliam, Zinhle Mthombothi, Tumelo Sereo, Mercy Kamupira, Sonia Botha, Koko Molema, Elizabeth Maseti, Marione Schönfeldt, Nicoletta Mabhena, Nishi Prabdial-Sing, Anne von Gottberg, Kerrigan McCarthy, Cari van Schalkwyk

**Affiliations:** a South African Centre for Epidemiological Modelling and Analysis (SACEMA), Stellenbosch University, Stellenbosch, South Africa; b UNICEF, South Africa; c Western Cape Department of Health, Expanded Programme on Immunisation, City of Cape Town, South Africa; d National Department of Health, Expanded Programme on Immunisation, Pretoria, South Africa; e Right to Care, Centurion, Pretoria, South Africa; f National Institute for Communicable Diseases, Division of the National Health Laboratory Service, Johannesburg, South Africa

**Keywords:** Poliovirus, Immunity, Zero-dose children, IPV, Vaccination, South Africa

## Abstract

In the context of polio eradication efforts, accurate assessment of vaccination programme effectiveness is essential to public health planning and decision making. Such assessments are often based on zero-dose children, estimated using the number of children who did not receive the first dose of the Diphtheria-Tetanus-Pertussis containing vaccine as a proxy.

Our study introduces a novel approach to directly estimate the number of children susceptible to poliovirus type 2 (PV2) and uses this approach to provide district-level estimates for South Africa of susceptible children born between 2017 and 2022. We used district-level data on annual doses of inactivated poliovirus vaccine (IPV) administered, live births, and population sizes, from 2017 through 2022. We imputed missing vaccination data, implemented flexible assumptions regarding dose distribution in the eligible population, and used estimated efficacy values for one, two, three, and four doses of IPV, to compute the number of susceptible and immune children by birth year. We validated our approach by comparing an intermediary output with zero-dose children (ZDC) estimated using data reported by WHO/UNICEF Estimates of National Immunization Coverage (WUENIC).

Our results indicate high heterogeneity in susceptibility to PV2 across South Africa’s 52 districts as of the end of 2022. In children under 5 years, PV2 susceptibility ranged from approximately 30 % in districts including Xhariep (31.9 %), Ekurhuleni (30.1 %), and Central Karoo (29.8 %), to less than 4 % in Sarah Baartman (1.9 %), Buffalo City (2.1 %), and eThekwini (3.2 %). Our susceptibility estimates were consistently higher than ZDC over the timeframe. We estimated that ZDC decreased nationally from 155,168 (152,737–158,523) in 2017 to 108,593 in 2021, and increased to 127,102 in 2022, a trend consistent with ZDC derived from data reported by WUENIC. While our approach provides a more comprehensive profile of PV2 susceptibility, our susceptibility and ZDC estimates generally agree in the ranking of districts according to risk.

## 1 Introduction

Poliomyelitis (polio) is a highly infectious disease that can lead to severe complications, including paralysis [1]. As outlined by the World Health Organization (WHO) and Global Polio Eradication Initiative (GPEI), vaccination is the primary strategy for polio eradication [2], [3], [4], [5]. South Africa has been free from local poliovirus transmission since 1989 and is currently certified as polio-free by the WHO [6]. However, maintenance of high vaccination rates is crucial to prevent resurgence, particularly in the context of recent wild poliovirus type 1 (WPV1) outbreaks in nearby countries, namely Malawi and Mozambique [7]. To support polio eradication efforts, South Africa includes poliovirus vaccines in the national expanded programme for immunization [8]. Infants receive their first dose of the oral poliovirus vaccine (OPV0) at birth; the second dose of OPV (OPV1) and the first dose of the inactivated poliovirus vaccine (IPV1), contained in the hexavalent *Hexaxim* vaccine [9], is administered at six weeks of age, with subsequent doses of IPV administered at ten weeks (IPV2), fourteen weeks (IPV3), and eighteen months (IPV4) [8]. All four doses of IPV were added to the South African vaccination schedule in 2009. The schedule has since remained the same, with the exception of the transition from Pentaxim (DTaP-IPV//HiB) to Hexaxim (DTaP-IPV-HB-Hib) in 2015.

IPV elicits a humoral response, which prevents poliovirus infections from progressing to disease, such as acute flaccid paralysis (AFP). OPV contains live-attenuated polioviruses and elicits both humoral and mucosal immune responses, thereby offering protection against infection and reducing transmission of polioviruses [10]. This, combined with the ability of OPV viruses to transmit to non-vaccinated individuals, makes OPV a key tool for eradicating polio and managing outbreaks. However, these same live attenuated viruses may, occasionally, through mutation and/or recombination, over time regain some or all of the transmissibility and neurovirulence properties of wild polioviruses. Such viruses are broadly referred to as vaccine-derived polioviruses (VDPVs) [1]. Following the eradication of WPV2, the remaining risk for AFP due to type 2 polioviruses comes from circulating vaccine derived poliovirus type 2 (cVDPV2). As a result, a global switch was implemented in April 2016 to replace trivalent OPV (tOPV) with bivalent OPV (bOPV), which excludes PV2, and the use of monovalent type 2 OPV (mOPV2) was limited to outbreak response efforts. The resulting immunity gap in younger populations, who have not received type 2 OPV and are thus capable of transmitting type 2 polioviruses, could amplify cVDPV2 outbreaks, increasing the risks associated with importation events. While these challenges are global, the geographical proximity of South Africa to countries such as Mozambique, Zimbabwe, and Botswana, where cVDPV2 has recently been detected, motivates our study focus on susceptibility to type 2 polioviruses [11].

Assessments of vaccine programme effectiveness provide key information both to local public health officials and to global organisations such as the WHO and GPEI [12]. In South Africa, as in many other countries, evaluation of vaccine programme performance involves analysis of coverage estimates for specific combinations of vaccines, and dropout rates between successive doses in a vaccination schedule. A popular metric of vaccination programme effectiveness used to monitor objectives of the WHO’s Immunization Agenda 2030 [13] is zero-dose children (ZDC), which represents the number of children born in a particular year who have not received any vaccinations from the country’s routine immunization schedule. This metric is estimated using a proxy of the number of children who did not receive the first dose of the Diphtheria-Tetanus-Pertussis (DTP) containing vaccine [14] which, in South Africa, is part of the IPV-containing *Hexaxim* vaccine and administered at six weeks of age. While simple to compute, existing zero-dose children estimates make strong assumptions about the distribution and uptake of subsequent vaccine doses, does not make use of all the available data, which often includes doses administered of all polio-containing vaccines in the schedule, and provides an indicator of susceptibility which is difficult to interpret mechanistically.

Vaccination programmes aim to increase population immunity against targeted diseases. While conventional metrics offer some insights via coverage estimates, estimates of population-level immunity are more mechanistically relevant to public health. Current methods for estimating population immunity across various diseases include the combination of vaccine efficacy data with records of individual-level vaccination histories [15], [16], the fitting of complex epidemic models [17], [18], [19], [20], [21], and serological surveys. We identified one current method for estimating mucosal immunity based on a combination of routinely collected proxy vaccination data and dates of supplemental immunization activities [22]. Recent data to support these approaches is not available in South Africa, where the last recorded poliomyelitis case was in 1989, the last poliovirus serosurvey was conducted in 1995, vaccination data is aggregated at the facility or district level, and type 2 OPV has not been administered since 2016. In this is study, we introduce a novel method for estimating population immunity that allows us to determine susceptibility among children born between 2017 and 2022 to PV2, using aggregated administrative data on vaccine doses administered at the district level in South Africa. We validate the zero-dose calculation embedded in our method through comparison with ZDC derived from data reported by WHO/UNICEF Estimates of National Immunization Coverage (WUENIC) [23].

## 2 Materials and methods

We estimate the numbers and proportions of children susceptible to PV2 among children born in each of South Africa’s 52 districts between 2017 and 2022, as of the end of 2022. Since bOPV, administered in place of tOPV since 2016, does not include PV2, we did not use OPV vaccination data and relied exclusively on IPV vaccination data. Our immunity estimation process involves imputing missing vaccination data, estimating the size of birth cohorts to whom vaccines are administered, implementing flexible assumptions regarding dose distribution, and using estimates of vaccine efficacies, to estimate the numbers of fully and partially susceptible and immune children by birth year.

### 2.1 Data sources

#### 2.1.1 Vaccine programme administrative data

We received district-level data from the National Department of Health (NDoH) which included information on the annual numbers of IPV 1, 2, 3, and 4 doses administered between 2017 and 2022.

Routinely collected data from NDoH was missing records for IPV2 doses administered between 2017 and 2019, for all 52 districts. We included a separate dataset on pentavalent IPV-containing vaccine doses administered after the transition to the hexavalent IPV-containing vaccine in 2015, to address the gaps where the primary dataset includes only the hexavalent vaccine. We associated doses of IPV4 administered in a particular year with children born in the previous year – as a result, children born in 2022 had not yet received their 18-month dose of IPV as of the end of 2022 (the reference date for our estimates).

#### 2.1.2 Demographics

To construct a comprehensive demographic profile, we integrated estimates from two sources:

• Mid-year district- and province-level population estimates of 0–4-year-olds between 2016 and 2022 as reported by the official source of South African population estimates, Statistics South Africa (Stats SA) [24].
• The Thembisa model [25]: Thembisa is a provincial-level demography and HIV model that is used by the NDoH for planning and budgeting the HIV response in South Africa. We used annual mid-year province-level estimates of zero-year-olds from the Thembisa model up to 2022 in combination with reported Stats SA population sizes to estimate district-level annual 0-year-olds (for details, see section 2.2).

#### 2.1.3 Estimates of zero-dose children

An intermediary output of our immunity estimation method, following bootstrapping of missing doses and the distribution of doses within birth cohorts, contains estimates of the numbers of children who received zero to four doses of IPV. We compared our intermediary estimates of children who received zero doses of IPV with estimates of the widely-used zero-dose children (ZDC) metric. The ZDC metric is calculated using a proxy definition of *children who did not receive the first dose of the DTP-containing vaccine* (which, in South Africa, contains IPV) and is based on data reported annually by WUENIC on DTP1 doses administered and numbers of children targeted for vaccination [23]. WUENIC estimates are reported as the number of ZDC per birth year.

### 2.2 Estimation of district-level annual 0-year-olds

Given the potential limitations of the available live births data from Stats SA, such as under-reporting, late registrations, and the unaccounted potential for movement between districts (as may occur when mothers and other caregivers seek healthcare), we estimated cohorts of 0-year-olds by combining the Stats SA district- and province-level data with provincial-level estimates from the Thembisa model. As shown in Eq. (1), to generate estimates of children less than one year of age in each district at the middle of each year from 2017 to 2022, we computed the proportions of Stats SA 0-4-year-olds in each district, per province, and multiplied these proportions with province-level estimates of zero-year-olds from the Thembisa model.

(1) $$D_{0}=SD_{0-4}/SP_{0-4}\times TP_{0}$$

where $D_{0}$ is district-level 0-year-olds, $SD_{0-4}$ is Stats SA district-level 0-4-year-olds, $SP_{0-4}$ is Stats SA province-level 0-4-year-olds, and $TP_{0}$ is Thembisa province-level 0-year-olds.

### 2.3 Imputation of missing vaccination data

We used a bootstrapping method to impute missing vaccination data. Data on IPV1, IPV3 and IPV4 were complete from 2017 through 2022; IPV2 data was only available for 2020 through 2022. We computed ratios of IPV2/IPV1 doses administered for 2020, 2021, and 2022. To impute the number of IPV2 doses administered in a given year, we randomly sampled once from these three IPV2/IPV1 ratios and multiplied the sampled ratio by the known number of IPV1 doses administered in that year. We repeated the sampling process 1,000 times to generate a range of estimates for the missing IPV2 doses. Details on an analysis of the number of samples required for the imputation procedure can be found in the supplement.

### 2.4 Redistribution of doses in districts with greater than 100 % coverage

Several districts reported more doses administered than the size of the estimated eligible birth cohort. This discrepancy suggests the possibility that children from neighbouring districts, where their births were officially registered, received vaccinations in these apparently over-vaccinated districts. To more accurately reflect the distribution of vaccine coverage by district of residence, we reallocated the remaining doses from districts and years where coverage exceeded 100 %, to neighbouring districts that reported less than 100 % coverage. The allocation was based proportionally on the numbers of unvaccinated children. Section 3 in the supplement shows the annual numbers of “extra doses” (where coverage was greater than 100 %) administered by district. To illustrate the impact of our assumption that “extra doses” were allocated to children living in neighbouring districts, we compared levels of susceptibility with and without redistribution, as shown in Table S1. Our method without redistribution excludes any “extra doses” from the analysis, which limits the vaccination coverage to a maximum of 100 % in each district.

### 2.5 Estimation of immunity levels

#### 2.5.1 Organised dose distribution

Based on the vaccination data described above, we estimated the number of children who received zero to four doses of IPV. We simulated the distribution of doses such that the maximum number of children born in a particular year are fully vaccinated. Remaining doses are distributed such that the maximum number of children received one fewer than the scheduled number of doses; this process is repeated until all doses have been allocated. This method accommodates children whose IPV doses received later than the recommended schedule were incorrectly recorded based on their age rather than their vaccination history, for example due to missing vaccine cards. Once doses are distributed, we compute the number of children who received zero doses for validation with estimates of zero-dose children derived from WUENIC, a publicly available data source [23]. To test the robustness of our dose distribution assumptions, we also simulated a scenario with random dose distribution (see supplementary materials section 2 for an explanation of the random dose distribution and examples of both methods).

#### 2.5.2 Vaccine efficacy and immunogenicity

We obtained estimates for the efficacy against PV2 of one (41 %) or two (80 %) doses of IPV, administered from 10 weeks of age, from a meta-analysis of seroprevalence studies [26]. To estimate the efficacy of three or four doses of IPV, we combined data from four studies that reported PV2 seroconversion rates following two and three doses of IPV [27], [28], [29], [30]. For each study, we extracted the proportion who gained immunity from the third dose (among those who were still susceptible after receiving two IPV doses). We fitted a beta distribution to these proportions, which ranged from 33 % to 100 %, using maximum likelihood estimation and used the mean of this distribution as our estimate of the per-dose efficacy of the third and fourth doses. To compute the efficacy of three doses, we multiplied our estimated efficacy of the third dose by the proportion susceptible after two doses (20 %) and added this to the two-dose efficacy. Similarly, the combined efficacy of four doses was estimated by multiplying the proportion susceptible after three doses by the efficacy of the fourth dose and adding this to the three-dose efficacy.

We applied our efficacy assumptions to the distributed IPV doses to compute the number of children with humoral immunity by birth year. We then calculated the proportion of immune individuals by birth year by dividing the estimated number of successfully immunised children by the number of allocated zero-year-olds, as detailed in section 2.2. We weighted the per-birth-year immunity estimates by population size to obtain an estimate of the proportion of zero- to five-year-olds, as of the end of 2022, with humoral immunity to PV2.

### 2.6 Outcomes: Zero-dose children and immunity levels

We estimate the numbers and proportions of children, by birth-year, from 2017 through 2022, who did not acquire humoral (or mucosal) immunity to PV2 – we refer to these children as susceptible children. We compare the districts identified with the highest and lowest risk using our (SACEMA’s) zero-dose children and susceptibility estimates in 2022.

## 3 Results

### 3.1 IPV efficacy estimates

We estimated a 78 % per-dose efficacy of IPV 3 and 4 against PV2. Using this estimate in combination with a previously estimated two-dose efficacy, we estimated three- and four-dose efficacies of 96 % and 98 % respectively. These estimates are consistent with the efficacies reported in other studies [31], [32].

### 3.2 Zero-dose children: Validation of intermediate outputs at the national level

**Fig. 1:**
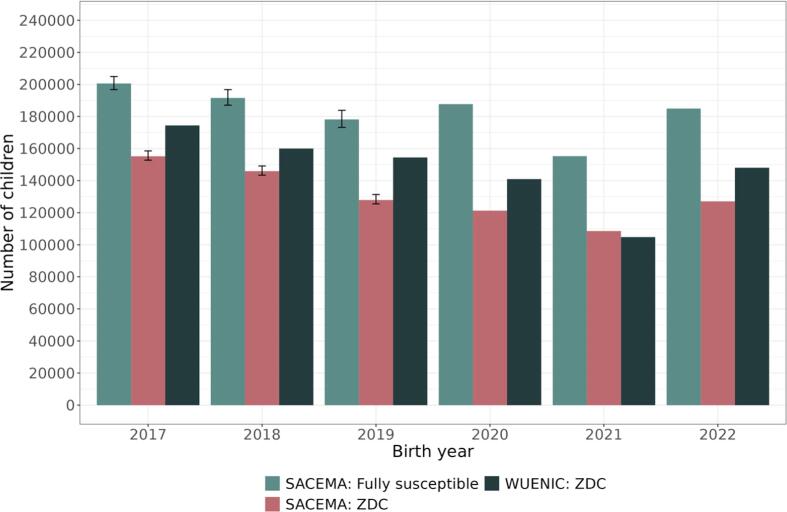
National-level comparison of SACEMA and WUENIC-derived zero-dose children (ZDC) estimates, alongside SACEMA estimates of numbers of children fully susceptible to PV2, by birth year. Black vertical bars represent the 2.5th and 97.5th quantiles of 1,000 bootstrap samples. As IPV data was complete for 2020–2022, bootstrapping was not necessary, hence the lack of variation in these years. There is a clear decreasing trend in estimates between 2017 and 2021, except for susceptibility estimates in 2020, and an increase from 2021 to 2022 in all three estimates.

We estimated that the annual number of children who did not receive a single dose of IPV in South Africa (SACEMA: ZDC) declined gradually from 155,168 (95 % bootstrap percentile interval: 152,737–158,523) in 2017 to 108,593 in 2021, with this figure increasing to 127,102 in 2022. Fig. 1 serves as a comparison between our estimates (SACEMA: ZDC) and with those derived from WUENIC data (defined as children who did not receive the first dose of the DTP-containing vaccine which, in South Africa, contains IPV). Estimates of zero-dose children using our dose distribution assumptions consistently decrease between 2017 and 2021 and increase in 2022; this trend aligns qualitatively with the trend in the WUENIC estimates. Estimates of susceptible children, made up of zero-dose children and those vaccinated but susceptible (due to imperfect vaccine efficacy), follow a similar trend, with the exception of 2020, which also showed the largest difference between estimates of zero-dose children and estimates of susceptible children. The number of children fully susceptible to PV2 is consistently higher than the numbers of zero-dose children.

### 3.3 Susceptible children: District-level estimates

#### 3.3.1 District-level estimates

Several districts with small numbers of zero-year-olds had high levels of susceptibility. In 2020, these include Xhariep, with 30.2 % of 2,910 zero-year-olds fully susceptible, and Central Karoo, with 27.6 % of 1,555 zero-year-olds fully susceptible. Several more populous districts, such as City of Ekurhuleni, also had high levels of susceptibility, with 30.9 % of 82,305 zero-year-olds fully susceptible. Figure S7 in the supplement outlines fully and partially susceptible children per birth-year between 2017 and 2022 for each district.

#### 3.3.2 National-level map of PV2 susceptibility

**Fig. 2:**
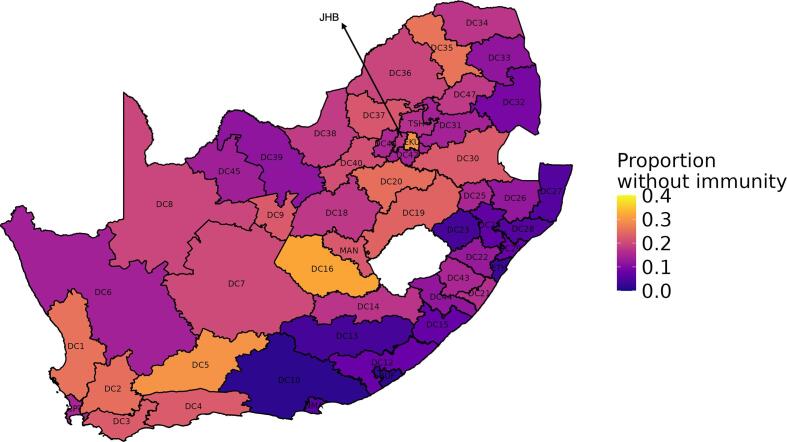
District-level proportions of fully susceptible children in the 0–4 age group in South Africa as of the end of end 2022. We estimate varying levels of susceptibility across districts, with higher levels of susceptibility in the northern and western regions of the country. In contrast, districts along the coast, particularly in the Kwa-Zulu Natal and Eastern Cape provinces, have lower levels of susceptibility.

Fig. 2 shows the proportions of 0-4-year-olds fully susceptible to PV2 for each of South Africa’s 52 districts as of the end of 2022 (corresponding to children born between 2018 and 2022). For this age group, susceptibility levels are highly heterogeneous across districts. The five districts with the highest susceptibility to PV2 are Xhariep (31.9 %), Ekurhuleni (30.1 %), Central Karoo (29.8 %), West Coast, and Capricorn (both 25.8 %). Ekurhuleni, the second highest-risk district, is located adjacent to City of Johannesburg, the country’s primary transportation hub. In contrast, districts such as Sarah Baartman, Buffalo City, and eThekwini, showcase commendable figures, each with less than 4 % estimated susceptibility to PV2 within this age group. Results for all 52 districts and a table with corresponding district codes and names are outlined in the supplement (Table S1 and Table S2).

#### 3.3.3 Comparison of high- and low-risk districts

We compared high- and low-risk districts using SACEMA’s estimates of the proportion of zero-dose children and susceptible children born in 2022. Table 1 shows the five highest and lowest risk districts. The ranking of districts was fairly consistent across both sets of estimates, although the proportion of zero-dose children were consistently lower than the proportion susceptible. This highlights that the zero-dose children metric may be underestimating the true susceptibility in the population. A comparison for the complete set of districts can be found in the supplement (Table S3).

**Table 1:** Highest and lowest risk districts for children born in 2022 according to SACEMA’s zero-dose children estimates and susceptibility estimates.

**District**	**SACEMA: Fully susceptible**		**SACEMA: Zero-dose children**	
	**Proportion**	**Rank**	**Proportion**	**Rank**
Central Karoo	32.1	1	27.6	1
Ekurhuleni	31.6	2	27.2	2
Xhariep	30.8	3	26.5	3
Cape Winelands	30	4	24.3	5
Gert Sibande	28.6	5	25.3	4
King Cetshwayo	4.1	48	0	49
Umkhanyakude	4	49	0	48
Buffalo City	4	50	0	45
Sarah Baartman	4	51	0	46
Dr Ruth Segomotsi Mompati	4	52	0	51

## 4 Discussion

No polio cases have been reported in South Africa since 1989, highlighting the significance of sustained vaccination efforts. However, the global transition from tOPV to bOPV in recent years leaves younger cohorts susceptible to PV2. Concern is compounded by recent evidence of polio transmission (cVDPV2) in neighbouring countries such as Mozambique, Zimbabwe, and Botswana. Within this context, our study provides a measure of the effectiveness of the vaccination programme against PV2 in recent years, and subsequently, PV2 immunity gaps in South Africa, highlighting not only the need to move beyond conventional zero-dose proxies when assessing population susceptibility, but also introducing a novel method for doing so.

Validation of our efficacy estimates and results at the national level is encouraging. Efficacy estimates for three and four doses of IPV align well with prior research [31], [32]. Both the general trend in and the numbers of zero-dose children at the national level, available as an intermediate output of our immunity estimation method, align well overall with estimates derived from WUENIC data, with a minor exception in 2020. Nationally, our susceptibility estimates decreased between 2017 and 2021, except for 2020, and increased from 2021 to 2022 (Fig. 1). While zero-dose children estimates reflect that IPV1 coverage was sustained during the COVID-19 pandemic, our estimates of susceptibility indicate a decline in coverage of subsequent IPV doses in 2020. While immunization programme performance seemed to improve in 2021, ZDC and susceptibility increased again in 2022. Unsurprisingly, given imperfect vaccine efficacy, we estimated that the number of children susceptible to PV2 was consistently higher than that of zero-dose children. This discrepancy will be larger in countries where fewer doses of IPV are administered. The degree of alignment in districts identified as highest and lowest risk using zero-dose children estimates versus susceptibility estimates depends on the consistency in vaccine drop-off rates (or their correlation with coverage).

A key advantage of directly estimating susceptible children is the use of all available data on doses administered, rather than relying only on the first dose of IPV. This is important because IPV, as with most vaccines, has limited efficacy. In the extreme case this would mean that a district with 100 % IPV1 coverage and zero doses of IPV2-4 administered would report zero ZDC when using the proxy measure, when in reality it would be overestimating the true immunity levels in the population. This is also critical to the international poliovirus elimination effort, since different vaccine schedules contain different numbers of IPV doses, which is not captured in ZDC estimates. In South Africa, for children born in 2022, we found that the high- and low-risk districts were consistent when using ZDC and susceptibility estimates, however, the proportions of ZDC were consistently lower than the proportion susceptible. Knowing which districts are most at risk in terms of susceptibility is crucial for allocation of potential interventions, given the high cost of remedial action and resource limitations.

Our method shows encouraging robustness to our dose distribution assumption, with estimates assuming random dose distribution aligning well with those based on organised dose distribution, despite starkly different implications for ZDC estimates (see supplementary materials section 2). This highlights the robustness of our method in comparison to the standard method for estimating ZDC, which includes a strong implicit assumption regarding dose distribution. Accurate estimates of susceptible or zero-dose children depend fundamentally on accurate estimates of population denominators, i.e. the children eligible to be vaccinated.

Overall, PV2 immunity levels are highly heterogeneous across South Africa. Ekurhuleni, a district with a large birth cohort, has a pronounced immunity gap which increases the risk of a cVDPV2 outbreak. A number of districts have alarmingly high levels of susceptibility including Xhariep, Ekurhuleni and Central Karoo with above approximately 30 % susceptibility in 0–4-year-olds. High-risk districts such as Capricorn and Waterberg (>20 % susceptibility), due to their proximity to Botswana, should be considered for targeted interventions such as catchup vaccination campaigns.

The disparities in PV2 immunity status cannot be viewed in isolation. The country's stark inequalities in household wealth, education and awareness, and urban vs rural healthcare infrastructure, have all been identified as risk factors for under-immunization [33], [34]. Addressing PV2 immunity gaps, therefore, requires a multi-pronged approach that not only focuses on immunization but also on addressing the root causes of these disparities.

## 5 Limitations

There are limitations which should be considered when interpreting our results. Firstly, we experienced various data constraints. Vaccination data was only available on an aggregated basis for each dose, which constrained our ability to determine exact vaccination combinations received by individual children. Additionally, our results may be limited due to district-level reporting inaccuracies in administrative vaccination data. We based our IPV efficacy assumptions on a meta-analysis that reported efficacies based on a schedule starting at 10 weeks of age, however, in South Africa, the IPV schedule begins at 6 weeks. Despite the slight misalignment between the schedules, we believe this meta-analysis provides the best available summary of IPV efficacy to inform our method. Questions regarding the reliability of the available district-level live births estimates meant we had to combine estimates from multiple sources to estimate the sizes of birth cohorts to which vaccines were administered. The results presented here do not include a rigorous handling of uncertainty, and while the data sources available do make this challenging, more could be done to improve this aspect of the methods. Our assumption regarding redistribution of doses in districts with greater than 100 % vaccine coverage has the potential to influence results in low-population districts that neighbour higher population districts with greater than 100 % coverage. Though this influence may reflect reality, due to people in rural areas crossing district borders while seeking healthcare in nearby cities and towns, the potential magnitude of this effect warrants further investigation into the dynamics of travel and healthcare seeking behaviour. It is also important to note that we did not differentiate between different doses in the immunization schedule, in essence this means that we assumed a constant efficacy for a set number of IPV doses administered to a child. Since evidence suggests that efficacy depends in part on the age of the child receiving a vaccine, this is an imperfect assumption. Finally, we recognise that the reliability of routinely collected immunization programme data on doses administered cannot be ascertained without additional study. Nonetheless, in many parts of the world, routinely collected immunization programme data, aggregated at a regional level, is the only consistently available source of data on population susceptibility to polioviruses.

### 5.1 Future work

This work forms part of a larger collection of studies. We will extend this methodology to include poliovirus types 1 and 3, as well as a wider age range target (0- to 85-year-olds and above). Should serological data become available, we will use the extended age targets to validate our immunity estimation method. We also intend to identify contexts where both immunization programme data and serological data are available, in order to validate our methods. We will also include data on supplementary immunization activities in South Africa and waning of immunity in our methods. This extended work will be used to initialise a poliovirus transmission model that was developed as part of the broader project. In addition, we aim to develop a flexible immunity estimation tool that can be used for similar pathogens in any geographical location.

## 6 Conclusion

We implemented a novel approach to estimating population susceptibility to PV2, providing estimates of susceptible children by birth year for children born between 2017 and 2022, and among 0–4-year-old children as of the end of 2022, for each of South Africa’s 52 districts. Our results indicate that susceptibility to PV2 is highly heterogenous between districts, ranging from approximately 30 % in the highest-risk districts to less than 4 % in others in 2022. While our observed high- and low-risk districts are consistent when using ZDC or susceptibility estimates, proportions of ZDC consistently underestimates the true susceptibility in the population. In summary, our method allows decision makers to utilize all of the routinely collected immunization programme data to provide a more robust and more directly interpretable indicator of population susceptibility to PV2.

## CRediT authorship contribution statement

**Lauren Brown:** Writing – review & editing, Writing – original draft, Visualization, Validation, Software, Resources, Project administration, Methodology, Investigation, Formal analysis, Conceptualization. **Jeremy Bingham:** Writing – review & editing, Writing – original draft, Visualization, Validation, Software, Resources, Project administration, Methodology, Investigation, Formal analysis, Conceptualization. **Juliet Pulliam:** Writing – review & editing, Writing – original draft, Supervision, Software, Resources, Project administration, Methodology, Investigation, Funding acquisition, Conceptualization. **Zinhle Mthombothi:** Writing – review & editing. **Tumelo Sereo:** Writing – review & editing. **Mercy Kamupira:** Writing – review & editing, Data curation. **Sonia Botha:** Writing – review & editing, Data curation. **Koko Molema:** Writing – review & editing, Data curation. **Elizabeth Maseti:** Writing – review & editing, Data curation. **Marione Schönfeldt:** Writing – review & editing, Data curation. **Nicoletta Mabhena:** Writing – review & editing, Data curation. **Nishi Prabdial-Sing:** Writing – review & editing, Data curation. **Anne von Gottberg:** Writing – review & editing, Supervision, Data curation. **Kerrigan McCarthy:** Writing – review & editing, Supervision, Methodology, Data curation. **Cari van Schalkwyk:** Writing – review & editing, Writing – original draft, Supervision, Methodology.

## Declaration of competing interest

The authors declare that they have no known competing financial interests or personal relationships that could have appeared to influence the work reported in this paper.

## Data Availability

Vaccination data can be requested on nhrd.health.gov.za. Demographics data are available from thembisa.org and statssa.gov.za. WUENIC data for validation is available from immunizationdata.who.int.
